# Analysis of prescription medication rules of traditional Chinese medicine for bradyarrhythmia treatment based on data mining

**DOI:** 10.1097/MD.0000000000031436

**Published:** 2022-11-04

**Authors:** Xujie Wang, Xuexue Zhang, Jiaxi Li, Biaoyan Hu, Jiwei Zhang, Wantong Zhang, Weiliang Weng, Qiuyan Li

**Affiliations:** a Xiyuan Hospital, China Academy of Chinese Medical Sciences, Beijing, China; b NMPA Key Laboratory for Clinical Research and Evaluation of Traditional Chinese Medicine, Beijing, China; c National Clinical Research Center for Chinese Medicine Cardiology, Beijing, China; d Shanxi University of Chinese Medicine, Taiyuan, China; e Peking University, Beijing, China.

**Keywords:** bradyarrhythmia, bradycardia, Chinese medicine, data mining, prescription medication rules

## Abstract

Multiple studies have revealed that Traditional Chinese Medicine (TCM) prescriptions can provide protective effect on the cardiovascular system, increase the heart rate and relieve the symptoms of patients with bradyarrhythmia. In China, the TCM treatment of bradyarrhythmia is very common, which is also an effective complementary therapy. In order to further understand the application of Chinese medicines in bradyarrhythmia, we analyzed the medication rules of TCM prescriptions for bradyarrhythmia by data mining methods based on previous clinical studies.

**Methods:**

We searched studies reporting the clinical effect of TCM on bradyarrhythmia in the PubMed and Chinese databases China National Knowledge Infrastructure database, and estimated publication bias by risk of bias tools ROB 2. Descriptive analysis, hierarchical clustering analysis and association rule analysis based on Apriori algorithm were carried out by Microsoft Excel, SPSS Modeler, SPSS Statistics and Rstidio, respectively. Association rules, co-occurrence and clustering among Chinese medicines were found.

**Results:**

A total of 48 studies were included in our study. Among the total 99 kinds of Chinese medicines, 22 high-frequency herbs were included. Four new prescriptions were obtained by hierarchical cluster analysis. 81 association rules were found based on association rule analysis, and a core prescription was intuitively based on the grouping matrix of the top 15 association rules (based on *confidence* level), of which Guizhi, Zhigancao, Wuweizi, Chuanxiong, Danshen, Danggui, Huangqi, Maidong, Dangshen, Rougui were the most strongly correlated herbs and in the core position.

**Conclusion:**

In this study, data mining strategy was applied to explore the TCM prescription for the treatment of bradyarrhythmia, and high-frequency herbs and core prescription were found. The core prescription was in line with the treatment ideas of TCM for bradyarrhythmia, which could intervene the disease from different aspects and adjust the patient’s Qi, blood, Yin and Yang, so as to achieve the purpose of treatment.

## 1. Introduction

Bradyarrhythmia is a common clinical arrhythmia. It is usually attributed to physiological conditions (e.g., well trained athletes), drug toxicity, genetic mutations, concurrent issues, or advanced age.^[1,2]^ Pathological changes that cause bradyarrhythmia are usually located in the sinus node, atrioventricular nodal tissue, atrial tissue, and the specialized conduction system.^[3]^ It can be further classified as sinus node dysfunction or sick sinus syndrome, atrioventricular block, and conduction tissue disease, depending on the location of the disease.^[4]^ Severe symptomatic bradyarrhythmia can lead to syncope and symptoms of heart failure, which can even be life-threatening.

The incidence of bradyarrhythmias is high in the elderly population, and a large cohort study has found that the incidence of bradyarrhythmias increases with age regardless of gender.^[5]^ With the acceleration of the aging process of the global population, the aging population base will be growing for a long time, and the number of patients with bradyarrhythmias will continue to increase.^[6]^ Therefore, bradyarrhythmia has received more attention around the world. The United States, Europe, China and other places are continuously updating their clinical guidelines or expert consensus for bradyarrhythmia, bringing new methods and strategies for the treatment of bradyarrhythmia.^[4,7,8]^

Clinical treatment of bradyarrhythmia mainly includes two types: drug therapy and pacing therapy. Pharmacotherapy is indicated for acute management due to drug toxicity, while pacemaker implantation is the essential therapy for the treatment of irreversible symptomatic bradyarrhythmias. In recent years, the use of pacemaker implantation has continued to grow worldwide. However, postoperative complications such as infection and heart failure, high cost and lifelong follow-up need are also the reasons why many people refuse or cannot afford pacemaker implantation.^[9–11]^ Such populations are at greater risk for severe complications and other accidents, and there is an urgent need to find alternatives for them.^[12]^

Traditional Chinese medicine (TCM) has a long history of treating bradyarrhythmias, which began in the Han dynasty in China (more than 2000 years ago). TCM has unique treatment advantages. It can be based on the development characteristics of the disease and from the concept of holism, to carry out syndrome differentiation and treatment, so as to adjust the Qi, blood, Yin and Yang inside and outside the human body, and achieve the therapeutic effect.^[13]^ For the pathogenesis of bradyarrhythmia, TCM theory is mostly considered to be due to blood stasis after Qi, Yang and Yin deficiency.^[14,15]^ TCM prescription has a beneficial intervention effect on bradyarrhythmia. The results of a meta-analysis^[16,17]^ and a network meta-analysis^[18]^ shows that a variety of TCM prescriptions can increase the heart rate, while effectively relieving the symptoms of patients with bradyarrhythmia. Therefore, this study searched the clinical research literature of TCM in the treatment of bradyarrhythmia since 2000, found common TCM prescriptions, used data mining methods for in-depth analysis, explored the therapeutic characteristics of TCM in the treatment of bradyarrhythmia, and provided data clues for related research.

The organization of this paper is arranged as follows.

Section 2 briefly discusses the application of data mining in TCM and related works. Section 3 presents sources of clinical data and their inclusion/exclusion criteria, as well as data mining methods of this manuscript. Section 4 gives the characteristics of the included studies and the results of data mining. Sections 5 and 6 discuss the data mining results, finally, summary and concluding remarks are given at the end along with references.

## 2. Related work

After thousands of years of development, TCM has accumulated a large number of various types of data. These complex data not only provide rich information, but also reflect obvious characteristics of massive information. Therefore, data mining technology is widely used in the field of Chinese medicine, and has achieved certain research results. Through data mining, text classification, cluster analysis and association analysis of TCM information will effectively extract the data mode in unstructured text, which can promote TCM informatization and structuring, so as to promote the modernization development of TCM.

At present, the application of data mining method in the research field of TCM is increasingly widespread. Zhang et al^[19]^ analyzed the prescription medication rule of TCM for diabetes treatment based on data mining. Zhang et al^[20]^ explored commonly used TCM formulas for the treatment of osteoporosis through data mining. Li et al^[21]^ based on data mining technology to optimize the process parameters of TCM. Dai et al^[22]^ studied the commonly used herbs of TCM for hyperlipidemia through clinical literature based on data mining technology. Wang et al^[23]^ excavated the core prescription of Weng Weiliang in the treatment of bradyarrhythmia based on complex network analysis. Lu et al^[24]^ identified herb combinations for Chinese herbal bath therapy for uremic pruritus using association rule analysis based on the Apriori algorithm. Hsieh et al^[25]^ also identified commonly used acupoint combinations for the treatment of chronic obstructive pulmonary disease using association rule analysis based on the Apriori algorithm. In addition, on the basis of data mining, some researches have analyzed the potential mechanisms of TCM prescription by network pharmacology and molecular docking. Ren et al^[26]^ used data mining and association networks to mine high-frequency Chinese herbs and formulas for the treatment of infectious diseases from ancient prescriptions, and then preliminarily revealed the molecular mechanisms through network pharmacology and molecular docking methods. Sun et al^[27]^ revealed the mechanisms of TCM in the treatment of Mycoplasma pneumoniae pneumonia based on data mining and systematic pharmacology. Qu et al^[28]^ revealed the medication rule of TCM in the treatment of premenstrual syndrome/premenstrual dysphoric disorder through network pharmacology and data mining approach.

The application of TCM therapy in bradyarrhythmias has gradually formed large-scale and has been continuously developed and deepened in recent years, and this type of research can reflect the characteristics and efficacy advantages of TCM.^[29]^ However, there is no study to integrate and analyze various clinical data on the treatment of bradyarrhythmias with Chinese medicine. Therefore, this study summarizes the clinical documents of various kinds of TCM treatment of bradyarrhythmias, and analyzed the high-frequency herbs and core prescription based on data mining, which can provide reference for the clinical treatment of bradyarrhythmia with TCM.

## 3. Material and Method

### 3.1. Clinical data sources and selection criteria

Systematically searched Pubmed (Medline) and Chinese databases China National Knowledge Internet database to identify randomized studies of TCM for bradyarrhythmia published from January 2000 to January 2022, with no language restriction. The full search strategy was employed combinations of medical subject headings terms and text words around “bradyarrhythmia,” “bradycardia,” “sick sinus syndrome,” “sinus node dysfunction,” “atrioventricular block,” “traditional Chinese medicine,” “Chinese medicine,” “herbal medicine,” “randomized controlled trial,” and “clinical trial.” Additional studies were derived from screening the reference lists of included randomized controlled trials and previous meta-annlysis studies.

#### 3.1.1. Inclusion criteria.

We aimed to comprehensively investigate the high-level clinical studies of Chinese medicine in the treating of bradyarrhythmias, so only randomized controlled trials in which TCM treatment shows exact therapeutic effect were included. In addition, included studies need to meet clinical diagnostic criteria for bradyarrhythmias. For the intervention methods, we hope to obtain all forms of herbal treatment: China’s National Medical Products Administration (NMPA)-approved patent Chinese medicine, classical formulas, self-designed formulas, single herb, and TCM-derived products.

Special note: For the same prescription appearing in different studies, we only included it once.

#### 3.1.2. Exclusion criteria.

Non-clinical studies: reviews and animal experiments. Non-randomized studies: retrospective studies, prospective studies, cohort studies, case-control studies, observational clinical trials or case reports. Non-oral decoction therapies: acupuncture, massage, moxibustion, external Chinese medicine, cupping, ear-point embedding beans, physical therapies, etc.

### 3.2. Quality assessment

For the included studies, the Revised Cochrane risk-of-bias tool for randomized trials^[30]^ was used to assess the risk of bias, including bias arising from the randomization process, bias due to deviations from intended interventions, bias due to missing outcome data, bias in measurement of the outcome, and bias in selection of the reported result. In this way, all articles selected for inclusion in the study were graded under the categories of low, some concerns, or high risk of bias.

### 3.3. Data mining

#### 3.3.1. Standardization of data.

In this study, all translations (Chinese-English) were mainly in accordance with the “WHO International Standard Terminologies on Traditional Medicine in the Western Pacific Region”^[31]^ and “International Standard Chinese-English Basic Nomenclature of Chinese Medicine.”^[32]^ Chinese medicines were standardized with reference to the Chinese Pharmacopoeia 2020 edition^[33]^ and unified as the official name.

#### 3.3.2. Data analysis.

All data were summarized into Microsoft Excel, and the bradyarrhythmia database of TCM was established. All data in this study were statistically analyzed using Microsoft Excel, SPSS Modeler, SPSS Statistics, and RStidio, respectively.

##### 3.3.2.1. Descriptive analysis.

The initial data were converted into transactional data, and the frequency of occurrence of each herb was subsequently calculated by an Excel pivot table,^[34]^ and we classified herbs with frequency greater than or equal to 5 as high-frequency drugs. Subsequently, the obtained data were imported into IBM SPSS Modeler 18.0 for co-occurrence analysis, with the upper limit of weak links set to 4 and the lower limit of strong links set to 9 to draw the TCM co-occurrence network diagram.

##### 3.3.2.2. Cluster analysis.

Cluster analysis can find the similarity between samples and samples by multivariate analysis.^[35]^ In this study, hierarchical clustering method was performed by IBM SPSS Statistics 26, and pearson correlation was used for similarity measure between variables. Through cluster analysis, TCM data were calculated according to a certain level, so that herbs with similar characteristics were gathered together to explore the relationship between TCM prescriptions for the treatment of bradyarrhythmia.

##### 3.3.2.3. Association analysis.

The association rules of Chinese herbal medicines in prescriptions were analyzed by the R software (RStidio) with an apriori algorithm in arules package. We performed association analysis by the Apriori algorithm and generated and visualized graphs by the R package “arulesViz.”

The resulting association rules are expressed in the form of implied expression X⇒Y, where X represents the antecedent item on the left hand side (*LHS*) and Y represents the consent item on the right hand side (*RHS*). Each association rule was evaluated using *support* and *confidence* levels.

“*Support*,” “*Confidence*,” and “*Lift*” are three standard metrics used to measure association between items (herbs) in the algorithm. The *support* degree represents the proportion of the number of occurrences of the associated data (*LHS* and *RHS*) appears in the total dataset (*N*). The *confidence* level is the conditional probability of data, which is the probability that *RHS* occurs after *LHS* occurs. The *lift* value indicates the probability of simultaneous *RHS* occurs in the occurs of *LHS*, as a ratio of the probability of overall occurrence of *LHS*. These three parameters statistically reflect herb compatibility. We set the threshold of the *support* degree to 0.1, and the *confidence* level to 0.6 to obtain the core herbs. The *support* degree, *confidence* level and *lift* value are used to evaluate each association rule, which can be calculated as follows:


$$\begin{array}{l} Support\left(X\Rightarrow Y\right)=Support(X\overset{}{\cup }Y)=\frac{Count(X\overset{}{\cup }Y)}{Count(N)} \end{array}$$ (1)

$$\begin{array}{l} Conf\;idence\left(X\Rightarrow Y\right)=\frac{Support(X\Rightarrow Y)}{Support(X)} \end{array}$$ (2)

$$\begin{array}{l} Lift\left(X\Rightarrow Y\right)=\frac{Conf\;idence(X\Rightarrow Y)}{Support(Y)} \end{array}$$ (3)

## 4. Result

### 4.1. Study characteristics and quality assessment

**Figure 1.**
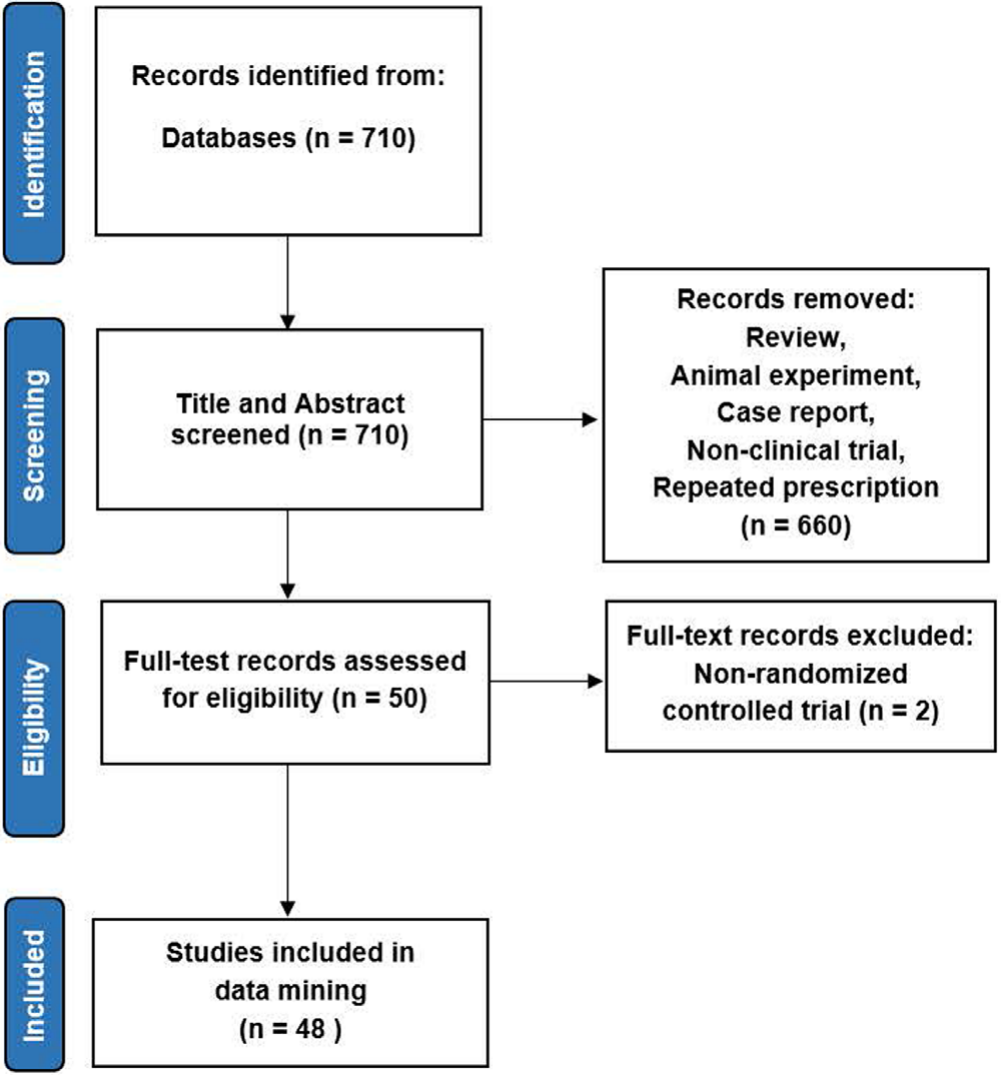
Flow chart.

We initially identified 710 potentially relevant publications through database searches. We screened the titles and abstracts of all articles. Fifty articles were selected for full-text review. Finally, 48 studies met our inclusion criteria and were included in the final analysis (Fig. 1). The characteristics of studies included in the study were shown in Table 1. The overall quality of the 48 studies were variable, with only two of them determined to be low risk of bias (Figure 1, Supplemental Digital Content, http://links.lww.com/MD/H777). The reason was that allocation concealment did not described in most studies and blinding did not performed.

**Table 1:** Characteristics of the included studies.

Study (year)	Study design	Inclusion criteria	Formula name	Herbs
Sun et al, 2020^[36]^	RCT	Bradyarrhythmia	Yixin Fumai granule	Renshen, Maidong, Wuweizi, Huangqi, Danshen, Chuanxiong
Guo et al, 2021^[37]^	RCT	Sinus bradycardia	Ershen Fumai capsule	Xiyangshen, Lurong, Danggui, Fuzi, Xianmao, Buguzhi, Guizhi, Houpo, Danshen, Longyanrou
Ma et al, 2020^[38]^	RCT	Bradyarrhythmia	Ginseng deer restorative decoction	Maidong, Hongshen, Gansong, Zhigancao, Dihuang, Lujiaojiao, Yinyanghuo, Tianzhuhuang, Dangshen, Danshen
Zou, 2020^[39]^	RCT	Bradyarrhythmia	Wenxin Wenlv decoction	Fuzi, Rougui, Ganjiang, Sangjisheng, Huanglian, Sanqi, Mudanpi, Danshen, Gansong, Zhigancao
Cao et al, 2019^[40]^	RCT	Bradyarrhythmia	Fumai capsule	Hongshen, Xiyangshen, Taizishen, Huangqi, Fuzi, Lurong, Zhigancao, Danshen, Chuanxiong
Fang et al, 2019^[41]^	RCT	Bradyarrhythmia	Liuwei Tongmai yin	Huangqi, Jixueteng, Baishao, Puhuang, Guizhi, Fangfeng
Zhu, 2019^[42]^	RCT	Bradyarrhythmia	Shenlu Fulyu decoction	Zhihuangqi, Lujiaojiao, Gualou, Hongshen, Yinyanghuo, Tianzhuhuang, Gansong, Taoren, Huanglian, Lulutong, Fuzi, Banxia, Zhishi, Paojiang, Zhigancao
Xu et al, 2019^[43]^	RCT	Bradyarrhythmia	Ningxin recipe II	Yinyanghuo, Fuzi, Huangqi, Maidong, Honghua, Danshen, Zhigancao
Shao et al, 2019^[44]^	RCT	Bradyarrhythmia	Yangxin Dingji capsule	Dihuang, Maidong, Hongshen, Dazao, Ejiao, Heizhima, Guizhi, Shengjiang, Zhigancao
Fan et al, 2018^[45]^	RCT	Bradyarrhythmia	Bindouling oral liquid	Hongshen, Huangqi, Fuzi, Yinyanghuo, Danshen, Shudihuang, Zhigancao
Sun et al, 2018^[46]^	RCT	Bradyarrhythmia	Yangxin Zhitong decoction	Huangqi, Taizishen, Fuling, Fushen, Yuanzhi, Banxia, Chenpi, Baiziren, Suanzaoren, Wuweizi, Danggui, Chuanxiong, Rougui, Zhigancao
Xu et al, 2018^[47]^	RCT	Bradyarrhythmia	Yangxinshi tablet	Huangqi, Dangshen, Danshen, Gegen, Yinyanghuo, Shanzha, Dihuang, Danggui, Huanglian, Yanhusuo, Lingzhi, Renshen, Zhigancao
Wei et al, 2018^[48]^	RCT	Bradyarrhythmia	Wenxin decoction	Maidong, Danshen, Xiebai, Taizishen, Chenpi, Wuweizi, Guizhi
Zhang et al, 2018^[49]^	RCT	SSS	Shexiang Baoxin pill	Rengong, Shexiang, Renshen, Rengong Niuhuang, Rougui, Suhexiang, Chansu, Bingpian
Zhang et al, 2017^[50]^	RCT	SSS	Zenglv Fumai granule	Renshen, Guizhi, Huangqi, Yinyanghuo, Danshen, Maidong, Chuanxiong, Huangjing
Ge et al, 2017^[51]^	RCT	SSS	Wentong Shengmai decoction	Guizhi, Maidong, Fuling, Baizhu, Hongshen, Wuweizi, Zhigancao
Li et al, 2017^[52]^	RCT	Bradyarrhythmia	Lyusheng Xinkang recipe	Guizhi, Zhigancao, Huangqi, Chaihu, Jiegeng, Shengma, Zhimu, Dangshen, Danggui, Baishao, Longyanrou, Shanzhuyu, Danshen
Li et al, 2017^[53]^	RCT	SSS	Ciwujia injection	Ciwujia
Liu, 2015^[54]^	RCT	Bradyarrhythmia	Yiqiwenyang decoction	Huangqi, Fuzi, Taizishen, Maidong, Xixin, Chuanxiong, Wuweizi, Renshen, Honghua, Danshen, Taoren, Zhigancao, Guizhi
Lin et al, 2015^[55]^	RCT	Bradyarrhythmia	Shengyu decoction	Huangqi, Dangshen, Shudihuang, Baishao, Danggui, Chuanxiong, Danshen, Guizhi
Liu et al, 2014^[56]^	RCT	SSS	Supplementing qi and activating yang prescription	Huangqi, Guizhi, Zhigancao, Danshen
Bai et al, 2014^[57]^	RCT	Bradyarrhythmia	Yiqizhumai recipe	Renshen, Huangqi, Shanzhuyu, Suanzaoren, Chishao, Danshen, Quanxie, Tubiechong, Gansong, Wuweizi
Wu et al, 2014^[58]^	RCT	SSS	Shenqi Yiqi Tongmai granule	Renshen, Fuzi, Huangqi, Mahuang, Maidong, Shuizhi, Danggui
Zhou et al, 2014^[59]^	RCT	Sinus bradycardia	Tongmai Yangxin pill	Zhigancao, Dangshen, Dihuang, Guizhi, Ejiao, Maidong, Dazao, Guijia, Wuweizi, Zhiheshouwu, Jixueteng
Liu et al, 2018^[60]^	RCT	SSS	Zishen Huoxue decoction	Fuzi, Huangqi, Sanqi, Zhigancao
Liu et al, 2012^[61]^	RCT	Bradyarrhythmia	Xinfukang	Fuzi, Mahuang, Xixin, Huangqi, Heshouwu, Yinyanghuo, Hongshen, Danshen
Liu et al, 2012^[62]^	RCT	Bradyarrhythmia	Modified Baoyuan decoction	Dangshen, Huangqi, Rougui, Zhigancao, Danggui, Baishao, Longyanrou, Shanzhuyu, Qianghuo
Peng et al, 2012^[63]^	RCT	Bradyarrhythmia	Ruanmai Xiaoban decoction	Fuzi, Jianghuang, Puhuang, Huzhang, Zexie, Heshouwu, Dihuang, Huangqi
Ren et al, 2012^[64]^	RCT	SSS	Shengxian decoction	Huangqi, Zhimu, Chaihu, Jiegeng, Shengma, Dangshen, Shanzhuyu, Guizhi, Zhigancao, Qianghuo
Cai, 2011^[65]^	RCT	Bradyarrhythmia	Shengmai decoction	Fuzi, Guizhi, Huangqi, Rougui, Danggui, Danshen, Maidong, Wuweizi, Zhigancao
Zhang, 2011^[66]^	RCT	Bradyarrhythmia	Yiqi Wenyang Huoxue Soup	Zhihuangqi, Taizishen, Fuzi, Xixin, Maidong, Wuweizi, Chuanxiong, Honghua, Taoren, Danshen, Guizhi, Zhigancao
Guo, 2011^[67]^	RCT	Bradyarrhythmia	Fumai decoction	Zhihuangqi, Renshen, Fuzi, Shudihuang, Yinyanghuo, Bajitian, Shanyao, Shanzhuyu, Fuling, Danggui, Gegen, Danshen, Gualou, Xiebai
Li et al, 2011^[68]^	RCT	SSS	Taoren Honghua Jian	Taoren, Honghua, Danggui, Chuanxiong, Chishao, Dihuang, Danshen, Yanhusuo, Xiangfu, Qingpi, Guizhi, Longgu, Muli, Gancao
Ma et al, 2010^[69]^	RCT	Bradyarrhythmia	Xinshen Shuangbu decoction	Zhihuangqi, Hongshen, Fuzi, Rougui, Xixin, Tusizi, Duzhong, Danshen, Shanzhuyu, Shanyao, Danggui, Zhigancao
Li, 2008^[70]^	RCT	SSS	Wenyang Fumai decoction	Fuzi, Yinyanghuo, Buguzhi, Dangshen, Danshen, Guizhi
Wang et al, 2008^[71]^	RCT	Sinus bradycardia	Qibo capsule	Huangqi, Fuzi
Wang et al, 2007^[72]^	RCT	Bradyarrhythmia	Shenglv decoction	Mahuang, Fuzi, Guizhi, Xixin, Rougui, Dangshen, Huangqi, Danggui, Baizhu, Xianmao, Gouqizi, Zhigancao
Deng, 2006^[73]^	RCT	Bradyarrhythmia	Shenglv granule	Dangshen, Huangqi, Fuzi, Mahuang, Taoren, Honghua, Shuizhi, Jiangxiang, Danggui, Xianmao, Yinyanghuo, Guizhi
Geng et al, 2006^[74]^	RCT	Sinus bradycardia	Fuxinmai capsule	Mahuang, Guizhi, Huangqi, Dangshen, Xixin, Danshen
Xu et al, 2003^[75]^	RCT	Bradyarrhythmia	Maisu capsule	Zhigancao, Renshen, Fuzi, Guizhi, Wuweizi, Maidong, Xiebai, Chishao
Liu, 2017^[76]^	RCT	Bradyarrhythmia	Shenxian Shengmai oral liquid	Hongshen Yinyanghuo Buguzhi Gouqizi Mahuang Xixin Danshen Shuizhi
Zhang et al, 2019^[77]^	RCT	Sinus bradycardia	Shensong yangxin capsule	Renshen, Maidong, Shanzhuyu, Danshen, Suanzaoren, Sangjisheng, Chishao, Tubiechong, Gansong, Huanglian, Wuweizi, Longgu
Wei et al, 2018^[78]^	RCT	Sinus bradycardia	Xinbao pill	Yangjinhua, Renshen, Rougui, Fuzi, Lurong, Bingpian, Rengong Shexiang, Sanqi, Chansu
Wei et al, 2012^[79]^	RCT	Bradyarrhythmia	Mahuangfuzixixin decoction	Mahuang, Xixin, Fuzi
Zhang et al, 2013^[80]^	RCT	Bradyarrhythmia	Zhigancao decoction	Zhigancao, Shengjiang, Guizhi, Renshen, Dihuang, Ejiao, Maidong, Huomaren, Dazao
Zheng et al, 2015^[81]^	RCT	Bradyarrhythmia	Shengmai injection	Hongshen, Maidong, Wuweizi
Liu et al, 2014^[82]^	RCT	Bradyarrhythmia	Ningxinbao capsule	Dongchongxiacao
Wang et al, 2011^[83]^	RCT	Bradyarrhythmia	Shenfu injection	Hongshen, Fuzi

RCT = randomized controlled trial, SSS = sick sinus syndrome.

### 4.2. Herbal frequency analysis

**Figure 2.**
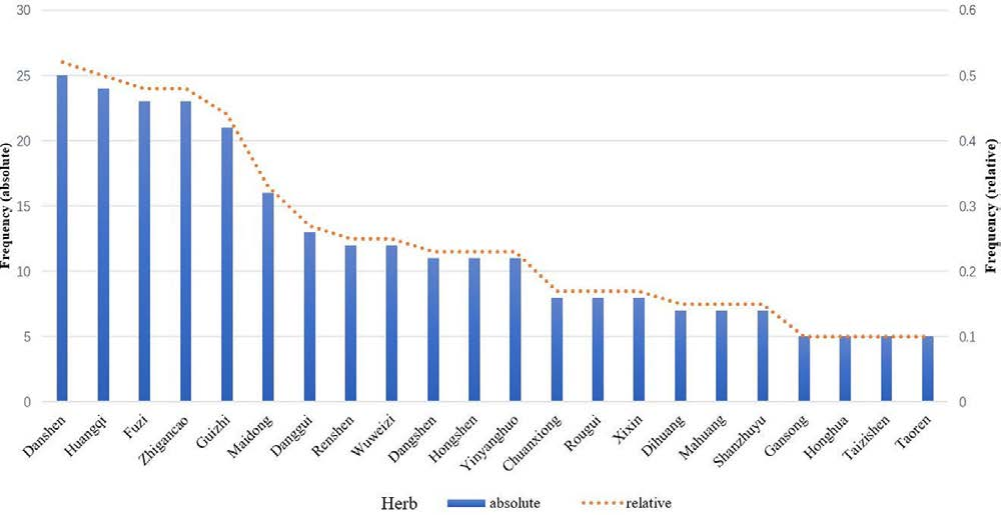
High-frequency Chinese medicines in prescriptions.

**Figure 3.**
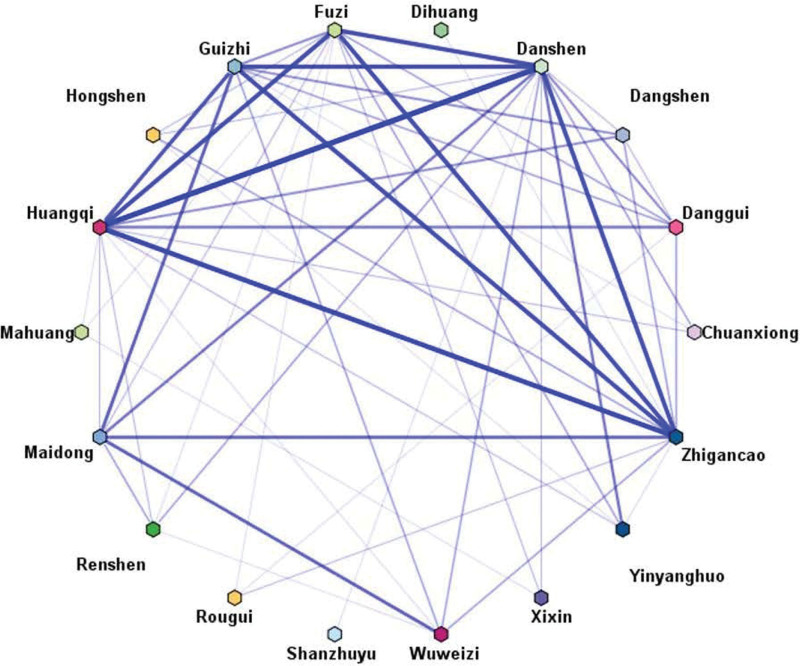
High-frequency herbs co-occurrence diagram.

A total of 99 kinds of herbs were obtained which were contained in 48 TCM prescriptions in 48 randomized controlled trials. High-frequency herbs (Frequency ≥ 5) were screened out through frequency analysis are shown in Figure 2. Information on the Latin scientific name, medicinal part, and specific frequency of herbal species are given in Table 2. Through frequency analysis, we found that the commonly used Chinese medicines for the treatment of bradyarrhythmia included: Danshen, Huangqi, Fuzi, Zhigancao, Guizhi, Maidong, Danggui, Renshen, Wuweizi, Dangshen, Hongshen, Yinyanghuo, Chuanxiong, Rougui, Xixin, Dihuang, Mahuang, Shanzhuyu, Gansong, Honghua, Taizishen, and Taoren, and the co-occurrence between herbs are shown in Figure 3.

**Table 2:** Basic information about high-frequency herbs.

No.	Chinese name	Latin name*	Medicinal part	Frequency
1	Danshen	*Salviae Miltiorrhizae Radix et Rhizoma*	Root, Rhizome	25
2	Huangqi	*Astraggli Radix*	Root	24
3	Fuzi	*Aconiti Lateralis Radix Praeparata*	Root	23
3	Zhigancao	*Glycyrrhizae Radix et Rhizoma Praeparata Cum Melle*	Rhizome	23
5	Guizhi	*Cinnamomi Ramulus*	Branch	21
6	Maidong	*Ophiopogonis Radix*	Root	16
7	Danggui	*Angelicae Sinensis Radix*	Root	13
8	Renshen	*Ginseng Radix et Rhizoma*	Root, Rhizome	12
8	Wuweizi	*Schisandrae Chinensis Fructus*	Fruit	12
10	Dangshen	*Codonopsis Radix*	Root	11
10	Hongshen	*Ginseng Radix et Rhizoma Rubra*	Root, Rhizome	11
10	Yinyanghuo	*Epimedii Folium*	Leaf	11
13	Chuanxiong	*Chuanxiong Rhizoma*	Rhizome	8
13	Rougui	*Cinnamomi Cortex*	Bark	8
13	Xixin	*Asari Radix et Rhizoma*	Root, Rhizome	8
16	Dihuang	*Rehmanniae Radix*	Root	7
16	Mahuang	*Ephedrae Herba*	Stem	7
16	Shanzhuyu	*Corni Fructus*	Fruit	7
19	Gansong	*Nardostachyos Radix et Rhizoma*	Root, Rhizome	5
19	Honghua	*Carthami Flos*	Flower	5
19	Taizishen	*Pseudostellariae Radix*	Root	5
19	Taoren	*Persicae Semen*	Seed	5

* The Latin names standardized by Chinese Pharmacopoeia (2020 edition).

### 4.3. Hierarchical cluster analysis

**Figure 4.**
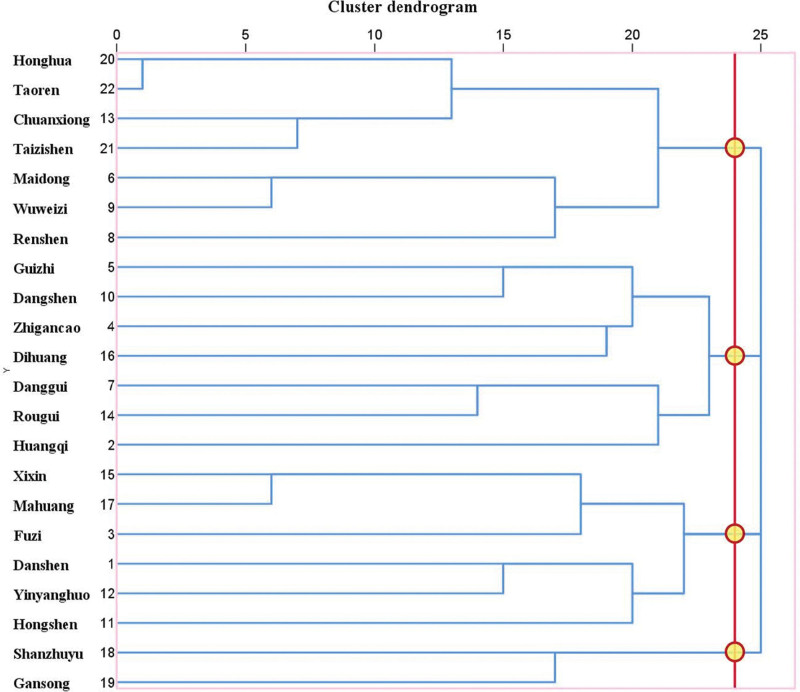
Hierarchical cluster analysis of high-frequency herbs.

The combination rule of different herbs can be determined by using hierarchical clustering method, which can classify herbs according to the relationship between herbs. The advantage of this method is that potential new prescriptions of Chinese medicines for the treatment of bradyarrhythmia can be found. In this study, we analyzed the selected high-frequency herbs. The TCM was stratified to obtain four different effective prescriptions based on TCM theory (Fig. 4).

### 4.4. Apriori algorithm-based association rule analysis

In order to further explore the compatibility between Chinese medicines for the treatment of bradyarrhythmia, we used Apriori algorithm-based association rule analysis to study the possible associations or connections between Chinese medicines.

**Figure 5.**
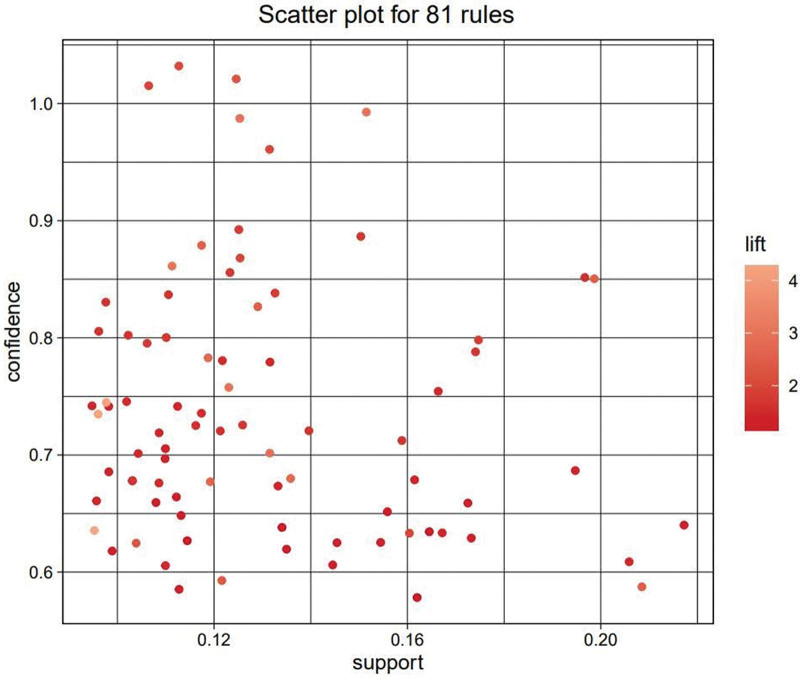
Overall scatter diagram of all rules.

We investigated 81 association rules based on the integrated data. The relationship among Chinese medicines were converted into the overall scatter diagram of all rules, with X axis as the *support* degree, Y axis as the *confidence* level, and the color of each association rule is determined by its *lift* value (Fig. 5). In this study, we found out the high-frequency of occurrence items in prescriptions based on mining association rules among herbs and filtered the top 15 rules based on the *confidence* level (Table 3).

**Table 3:** Top 15 Apriori algorithm-based association rules of herbs.

No.	Association rules	Support	Confidence	Lift	Count
1	{Danggui, Rougui} => {Zhigancao}	0.104167	1	2.086957	5
2	{Chuanxiong, Guizhi} => {Danshen}	0.104167	1	1.92	5
3	{Danggui, Dangshen} => {Huangqi}	0.125	1	2	6
4	{Guizhi, Wuweizi} => {Maidong}	0.145833	1	3	7
5	{Maidong, Wuweizi, Zhigancao} => {Guizhi}	0.125	1	2.285714	6
6	{Guizhi, Wuweizi, Zhigancao} => {Maidong}	0.125	1	3	6
7	{Chuanxiong} => {Danshen}	0.145833	0.875	1.68	7
8	{Wuweizi, Zhigancao} => {Maidong}	0.125	0.857143	2.571429	6
9	{Danshen, Wuweizi} => {Maidong}	0.125	0.857143	2.571429	6
10	{Guizhi, Wuweizi} => {Zhigancao}	0.125	0.857143	1.78882	6
11	{Wuweizi, Zhigancao} => {Guizhi}	0.125	0.857143	1.959184	6
12	{Danggui, Zhigancao} => {Huangqi}	0.125	0.857143	1.714286	6
13	{Guizhi, Maidong, Wuweizi} => {Zhigancao}	0.125	0.857143	1.78882	6
14	{Wuweizi} => {Maidong}	0.208333	0.833333	2.5	10
15	{Rougui, Zhigancao} => {Danggui}	0.104167	0.833333	3.076923	5

**Figure 6.**
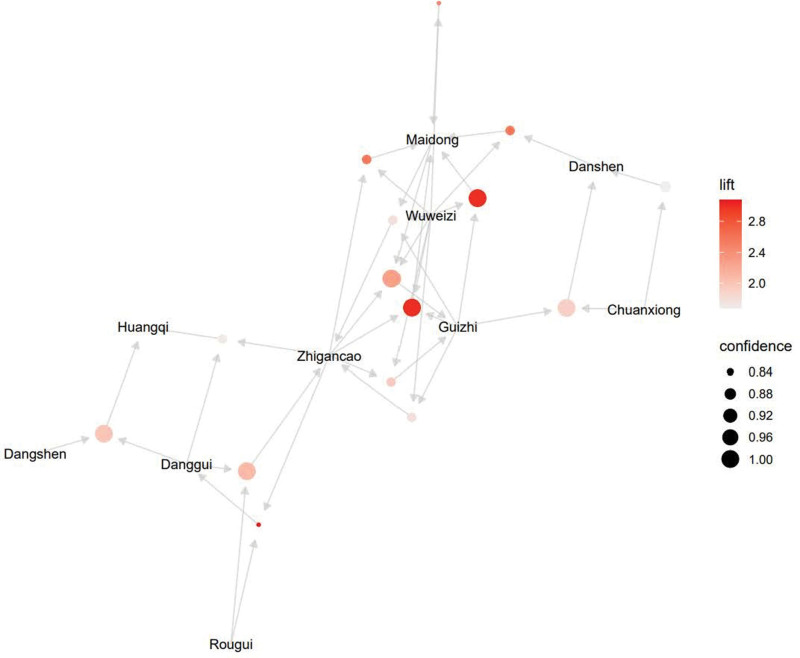
Overall scatter diagram of top 15 association rules.

With respect to grouped sets of items, we used association network graph based on color or size visualization to achieve the visualization of herbs compatibility relations (Fig. 6). These features are presented intuitively based on a grouping matrix of 15 association rules. This figure provides a clear representation of association rules and avoids cluttered presentation. It could be seen that Guizhi, Zhigancao, Wuweizi, Chuanxiong, Danshen, Danggui, Huangqi, Maidong, Dangshen, Rougui were the most strongly correlated herbs and in the core position.

## 5. Discussion

In recent years, with Professor Tu Youyou becoming the first Nobel Prize winner in Physiology or Medicine in China, TCM has attracted more and more attention from the international community.^[84]^ In March 2022, a meeting report^[85]^ released by the World Health Organization (WHO) also indicated that TCM was both safe and beneficial when combined with conventional antiviral medicine for coronavirus disease 2019 treatment. A variety of TCM formulas and pharmaceutical extracts have been discovered to show potential in preventing coronavirus disease 2019.^[86,87]^ Traditional, complementary, and integrative medicine can make important contributions to health management, disease prevention, and treatment of major diseases. Like artemisinin, extracted from Chinese herb Qinghao (Artemisiae Annuae Herba), Chinese medicines and its derivatives are generally inexpensive, while having a good therapeutic effect. Therefore, TCM treatment of bradyarrhythmia requires in-depth mining and analysis, which can benefit patients with bradyarrhythmia and allow such population to have more treatment options.

The nature and function of Chinese medicine formed the fundamental basis for analyzing Chinese herbs as well as its clinical applications. In this study, a variety of statistical methods were used to perform a holistic analysis of Chinese medicines in prescriptions for the treatment of bradyarrhythmia. Through descriptive statistical analysis of TCM data in prescriptions for the treatment of bradyarrhythmia, we found high-frequency Chinese medicines in TCM prescriptions, of which Danshen, Danggui, Chuanxiong, Honghua, and Taoren were commonly used in clinical practice for activating blood and resolving stasis, Huangqi, Zhigancao, Renshen, Dangshen, and Taizishen were commonly used in clinical practice for tonifying Qi, Fuzi, Guizhi, Hongshen, Yinyanghuo, Rougui, Xixin, and Mahuang were commonly used in clinical practice for warming Yang, Maidong, Wuweizi, Dihuang, Shanzhuyu were commonly used in clinical practice for nourishing Yin, and Gansong is a herb for moving Qi.

The application of hierarchical cluster analysis allowed us to find four potential TCM prescriptions for the treatment of bradyarrhythmia among high-frequency herbs. From Figure 4, it can be seen that the number of herbs in the first three prescriptions was more appropriate, while the fourth prescription only includes two herbs, Shanzhuyu and Gansong. Due to the small number of herbs, we did not consider it as a complete prescription. However, according to long-term clinical experience of TCM, we found that if the herb of prescription 4 and the herb of prescription 1 were combined into one prescription (prescription 5), the drug composition of this prescription was very close to the Chinese patent medicine “Shensong yangxin capsule (SSYX),” which has the functions of replenishing Qi and nourishing Yin, and activating blood and resolving stasis. Long-term SSYX treatment has been shown to restore calcium homeostasis and increase heart rate in the rabbit model of bradycardia by enhancing the expression of ryanodine receptor 2, sarcoplasmic/endoplasmic reticulum Ca^2+^ATPase 2 and voltage-dependent anion-selective channel.^[88,89]^ From the analysis of the medicinal efficacy of each herb in prescription 2, its clinical effect was similar to that of the classical prescription “Zhigancao decoction” in *Treatise on Cold Pathogenic and Miscellaneous Disease,*^[90]^ which has the effects of nourishing Yin and blood, warming Yang and replenishing Qi. The efficacy of prescription 3 is close to that of the classical prescription “Mahuangfuzixixin Decoction” and the Chinese patent medicine “Shenxian Shengmai oral liquid (SXSM),” which has the effect of warming Yang and activating blood. Studies have shown that SXSM can affect in calcium handling and signaling, promote myocardial oxidative phosphorylation and tricarboxylic acid cycle, improve adenosine triphosphate production, and stimulate sympathetic transmission by upregulating β1-adrenoceptor, increasing acetylcholinesterase and reducing nicotinic receptors, thus increasing heart rate.^[91,92]^

Through association rule analysis based on Apriori algorithm, we found 81 rules, which can show the correlation of herb compatibility. Based on the definition of *confidence* level, we believe that the compatibility of herbal medicines with low *confidence* has low reference value for clinical practice, only the first 15 association rules are selected, so as to summarize the core prescription of Chinese medicines for the treatment of bradyarrhythmia. According to the theory of TCM, the core prescription has the effects of warming Yang and activating blood, replenishing Qi and nourishing Yin, which may be potentially useful in the treatment of bradyarrhythmia. Further clinical and pharmacodynamic experiments are required to validate the observation results of this study and explore the main components and mechanism of action of the core prescription.

## 6. Conclusions

The data mining strategy was applied in this study to explore Chinese medicine prescriptions for the treatment of bradyarrhythmia, high-frequency herbs were selected, and then three potential prescriptions were found by hierarchical cluster analysis, and one core prescription was excavated by association rule analysis. The compatibility of herbal medicines in the above four prescriptions is consistent with the therapeutic strategy of TCM for bradyarrhythmia, which can intervene the disease from different aspects and adjust the Qi, blood, Yin and Yang of patients, so as to achieve the purpose of treatment. Data mining can combine a variety of statistical methods to analyze TCM clinical research data and discover more therapeutic strategies from different perspectives. However, such analysis is limited to the secondary analysis of existing literature data, and could not be deeply explored in terms of drug efficacy and mechanism of action. Therefore, the research results still need to be verified by clinical and pharmacodynamic studies.

## Author contributions

**Conceptualization:** Xujie Wang, Wantong Zhang, Weiliang Weng, Qiuyan Li.

**Data curation:** Xujie Wang, Xuexue Zhang, Biaoyan Hu.

**Formal analysis:** Xujie Wang, Biaoyan Hu.

**Methodology:** Xujie Wang.

**Software:** Xujie Wang, Xuexue Zhang.

**Supervision:** Weiliang Weng, Qiuyan Li.

**Visualization:** Xujie Wang.

**Writing – original draft:** Xujie Wang, Xuexue Zhang, Jiaxi Li.

**Writing – review & editing:** Jiwei Zhang, Wantong Zhang, Weiliang Weng, Qiuyan Li.

## Supplementary Material


