# Supplementary figures and images for: Analysis of prescription medication rules of traditional Chinese medicine for bradyarrhythmia treatment based on data mining
Source: Medicine (Baltimore). 2022 Nov 4;101(44):e31436. doi: 10.1097/MD.0000000000031436 (PMC9646641; doi:10.1097/MD.0000000000031436)

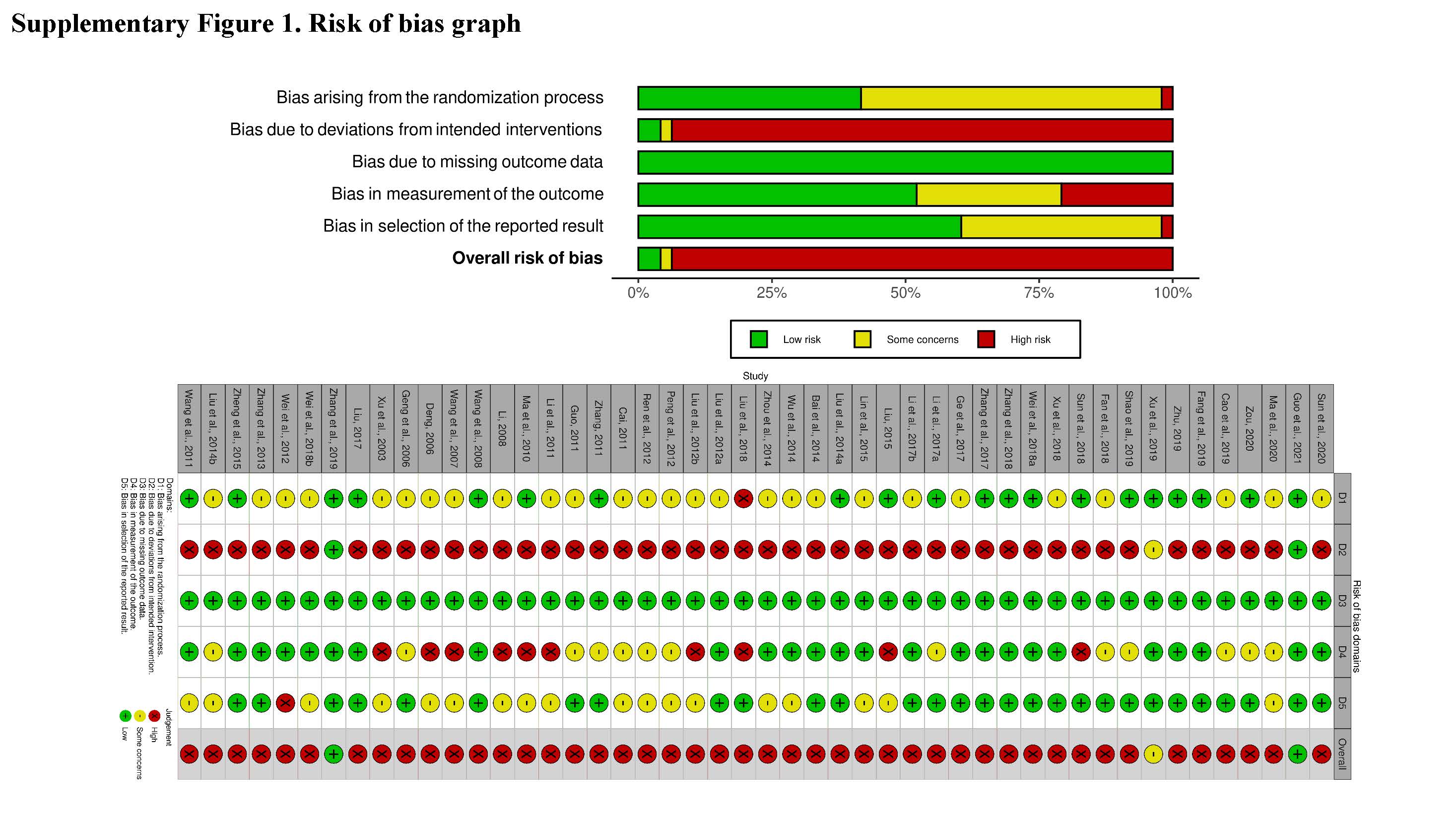

Supplement: Supplementary file 1 [file medi-101-e31436-s001.tif]
